# Anaesthetic Techniques for Cardiac Ablation—A Scoping Review Protocol

**DOI:** 10.1111/aas.70054

**Published:** 2025-05-07

**Authors:** Mathilde Bang Fredensborg, Sebastian Bach Fiege, Ann M. Møller

**Affiliations:** ^1^ Department of Anaesthesiology Herlev Anaesthesia Critical and Emergency Care Science Unit (ACES), Copenhagen University Hospital—Herlev Hospital Herlev Denmark; ^2^ Department of Clinical Medicine Faculty of Health Sciences, University of Copenhagen Copenhagen Denmark; ^3^ Department of Anaesthesiology Herlev and Gentofte Hospital Gentofte Denmark

**Keywords:** anaesthesia, cardiac ablations, cardiac arrhythmias, conscious sedation, deep sedation, sedation

## Abstract

**Background:**

Cardiac arrhythmias, particularly atrial fibrillation, are highly prevalent and represent a growing global health burden. Cardiac ablation is a commonly used procedure for the treatment of cardiac arrhythmias, often requiring anaesthesia to ensure patient safety and procedural success. Despite its widespread use, there is no consensus on the optimal anaesthetic strategy, and existing literature mainly addresses isolated techniques or specific populations. The aim of this scoping review is to systematically map the current evidence on anaesthetic methods used during cardiac ablation procedures.

**Methods:**

A scoping review will be conducted in accordance with the Preferred Reporting Items for Systematic Reviews and Meta‐Analyses extension for scoping reviews (PRISMA‐ScR) guidelines. A comprehensive search strategy will be applied across multiple databases, including Medline, EMBASE, Cochrane, Scopus and Google Scholar. Eligible studies will include all research on adult patients undergoing cardiac ablation, focusing on any anaesthetic or sedative techniques used. Study selection and data extraction will be performed by independent reviewers, and results will be summarized descriptively.

**Results:**

In the final review, findings will be presented through a descriptive and narrative summary, supplemented by tables, figures and graphs where applicable.

**Conclusion:**

This scoping review will provide a structured overview of the landscape of anaesthesia methods in cardiac ablations. The findings will help uncover knowledge gaps and inform future research and clinical decision‐making regarding anaesthesia strategies in the management of cardiac arrhythmias.

## 1 Introduction

### 1.1 Rationale

Cardiovascular diseases remain a leading cause of morbidity and mortality worldwide with an estimated 17.9 million deaths annually [1]. These conditions place a significant burden on healthcare systems, affect the economy of society and profoundly impact patients' lives [2, 3].

Among cardiovascular disorders, cardiac arrhythmias play a huge role due to their high prevalence, associated complications and the degree of patient impairment they can cause.

Cardiac arrhythmias are becoming increasingly common globally [3]. The most prevalent form is atrial fibrillation (AF) with a prevalence exceeding 33 million worldwide. AF is a major risk factor for stroke, heart failure and increased mortality, making it a central focus point in cardiovascular medicine [4, 5].

Treatment options for arrhythmias include pharmacological treatment, DC cardioversion and cardiac ablations. Cardiac ablation is a widely used intervention for managing various cardiac arrhythmias, including AF and ventricular tachycardia. These procedures often require anaesthesia or sedation to ensure patient comfort, optimize procedural conditions and minimize complications.

A range of anaesthetic techniques may be employed, including general anaesthesia (GA), deep sedation (DS) or conscious sedation (CS). Anaesthesia and sedation are essential components of cardiac ablations. However, the optimal anaesthesia strategy remains a subject of debate [6, 7].

The existing literature has mainly focused on the safety and efficacy of specific anaesthetic techniques in cardiac procedures by looking at single modalities or specific patient populations. While these studies provide valuable insights in anaesthetic agents and techniques, a comprehensive overview of the anaesthetic methods used for cardiac ablations has yet to be conducted.

This scoping review aims to address this gap by systematically mapping the available evidence on anaesthetic methods used during cardiac ablations. Specifically, it will identify the range of anaesthetic approaches employed, associated complications, effects on recovery time, patient and operator satisfaction, geographic variation and outcome measures used to assess the anaesthetic quality.

### 1.2 Objectives

The main objective of this scoping review is to provide an overview of existing evidence on anaesthetic techniques used in cardiac ablations. The focus will be on adverse events, recovery, patient and operator satisfaction, geographic variation and outcome measures used to evaluate the quality of anaesthesia. This scoping review's aim is to map existing literature, identify gaps and serve as a foundation for future research.

1. Which anaesthetic techniques are currently used for cardiac ablations?
2. What are the reported complications or adverse events associated with different anaesthetic approaches for cardiac ablations?
3. How do the anaesthetic techniques facilitate post‐procedural recovery?
4. Are there differences in patient satisfaction or operator satisfaction depending on the anaesthesia method used?
5. Are there variations in anaesthesia practices across geographic regions or healthcare settings?
6. What are the most reported outcome measures used to assess anaesthesia effectiveness and quality in these procedures?

## 2 Methods

This protocol has been developed following the Preferred Reporting Items for Systematic Review and Meta‐Analysis Protocols (PRISMA‐P). The scoping review will be carried out in alignment with the Preferred Reporting Items for Systematic Reviews and Meta‐Analyses extension for Scoping Reviews (PRISMA‐ScR) checklist.

### 2.1 Eligibility Criteria

#### 2.1.1 Population

Adult patients (age over 18) undergoing cardiac ablation procedures.

#### 2.1.2 Concept

All kinds of anaesthesia and sedative techniques used for cardiac ablation, including GA, DS and CS.

#### 2.1.3 Context

Cardiac ablation procedures are performed in hospitals, clinics or similar healthcare facilities.

The review will include all primary studies, where the primary outcome involves the anaesthetic technique being investigated, irrespective of study design, publication year, setting and duration. The geographic location will not be a limiting factor for inclusion. We will be excluding editorials and letters, and anything with patients below the age of 18.

### 2.2 Information Sources

A thorough literature search will be conducted across various databases including MEDLINE, EMBASE, Cochrane Central Register of Controlled Trials, Scopus and Google Scholar. Additionally, we will scan the reference list of the included studies to ensure all relevant studies have been assessed.

### 2.3 Search Strategy

The search strategy will be developed with the help of an information specialist with experience in scoping reviews. An initial search strategy for MEDLINE is outlined in Appendix B. Any additional relevant key words identified during the process will be integrated. No search limits or filters such as language, publication year or study design will be used.

### 2.4 Study Records

All retrieved literature will be uploaded to a web‐based screening and data extraction tool. Duplicate studies will be removed. At least two independent reviewers will evaluate the eligibility and inclusion of all studies, resolving any discrepancies through discussion or by consulting an impartial third party.

Data charting will be performed by two independent reviewers using a predefined data charting form. The form may be refined if necessary. Any modifications will be clearly documented in the final review.

### 2.5 Data Items

The extracted data variables will include the following: study characteristics (author(s), year, aim, country, etc.), population (age, gender, sample size), procedure (type, diagnosis, duration) and anaesthesia (type, medications, complications, etc.). A preliminary data extraction form is provided in Appendix A.

### 2.6 Outcomes and Prioritization

The primary outcome will be to identify anaesthetic techniques in cardiac ablations. Secondary outcomes include complications (respiratory and hemodynamic instability, etc.), recovery time and emergence. Furthermore, patient and operator satisfaction, the geographic variation in choice of anaesthesia and outcome measures used to evaluate the anaesthetic quality will be assessed.

### 2.7 Data Synthesis

Data will be summarized using descriptive and narrative analysis. Descriptive analysis will capture study characteristics and anaesthesia‐related variables, while narrative synthesis will explore trends, differences and themes across studies. Where appropriate, summary statistics, tables and figures will be used to support data presentation.

### 2.8 Quality of Evidence

Formal risk of bias assessment will not be conducted, consistent with scoping review methodology. However, a narrative appraisal of the overall quality and heterogeneity of the included evidence will be provided in Section 3.

## 3 Discussion

This scoping review aims to provide a comprehensive overview of the anaesthetic techniques used for cardiac ablations. It will map out the various methods, geographic variation, associated complications, post‐procedure recovery, differences in patient or operator satisfaction and the most commonly used outcomes to evaluate the quality of anaesthesia.

This scoping review will have several strengths, including a predefined and published protocol. Furthermore, it will be conducted in accordance with the PRISMA‐ScR. Additionally, the scoping review has been carried out using a broad and systematic search strategy and will include a wide range of study types.

However, there are limitations; a risk of bias assessment will not be conducted for the individual studies [8].

## 4 Conclusion

This scoping review will give an extensive overview of the anaesthetic approaches used for cardiac ablations. It will examine the associated complications, recovery, patient and operator satisfaction, variation in practice and highlight the most reported outcome measures used for assessing the quality of anaesthesia.

The review is being undertaken as part of a broader effort to explore and evaluate anaesthetic strategies for cardiac ablation. The findings will be used to identify research gaps, inform the design of future clinical studies and ultimately contribute to improved clinical decision‐making regarding anaesthesia in this field.

## Author Contributions

Developed the protocol and wrote the initial draft of the manuscript: Mathilde Bang Fredensborg. Reviewed the manuscript and provided guidance throughout the project: Sebastian Bach Fiege. Provided supervision and guidance throughout the project as a primary advisor: Ann M. Møller. All authors reviewed and approved the final version of the manuscript.

## Conflicts of Interest

The authors declare no conflicts of interest.

## Data Availability

No new data will be created or analysed in this study. Data sharing is not applicable to this article. .

## Appendix A See Table A1

**TABLE A1:** Will be used for the preliminary data extraction form.

Study characteristics	Database
	Journal
	Author(s), year
	Title
	Country
	Type of study
	Aim
Population	Age
	Gender
	Sample size
	Diagnosis
Procedure	Ablation type
	Duration
Anaesthesia	Type
	Medications used
	Complications
	Emergence and recovery time
	Patient and operator satisfaction
	Outcome measures used to assess anaesthesia quality
Outcomes/results	

## Appendix B See Table B1

**TABLE B1:** Search threat from MEDLINE the 3 April 2025.

#	Query	Results from 3 April 2025
#1	(ablat* adj3 techniq*).mp.	9114
#2	ablation techniques/or cryosurgery/or radiofrequency ablation/or catheter ablation/or maze procedure/	60,389
#3	((Catheter* adj2 ablat*) or (Radiofrequenc* adj2 ablat*) or Cryoablat* or (pulse* adj2 field* adj2 ablat*) or (Pulmon* adj2 Vein* adj2 isolat*) or RFA or PFA).mp.	68,971
#4	(An?esth* or Anaesth* or Anesth*).mp.	526,078
#5	sedat*.mp.	95,463
#6	exp anesthesia/or conscious sedation/or deep sedation/	220,291
#7	#4 OR #5 OR 6	607,292
#8	exp Arrhythmias, Cardiac/	248,934
#9	(arrhythmi* or (Atria* adj fib*) or (Atria* adj flutter*) or (Atrioventricular adj3 tachycardi*) or Wolff‐Parkinson‐White syndrome or (Ventric* adj2 tachycardi*) or Ventric* ectopic* beat* or Premature ventric* contraction* or Sinus node reentr* tachycardi*).mp.	289,360
#10	#8 OR #9	349,322
#11	#1 OR #2 OR #3	84,678
#12	#7 AND #10 AND #11	774
